# Risk assessment of pre-excitation: Atrial fibrillation versus atrial flutter

**DOI:** 10.1016/j.hrcr.2022.10.003

**Published:** 2022-10-07

**Authors:** Jonathan Uniat, Michael J. Silka

**Affiliations:** Division of Cardiology, Children’s Hospital Los Angeles, Keck School of Medicine, University of Southern California, Los Angeles, California

**Keywords:** Pre-excitation, Wolff-Parkinson-White syndrome, Sudden death, Ablation, Risk stratification

**Key Teaching Points**

• Owing to concealed conduction, the shortest pre-excited R-R intervals during atrial fibrillation and atrial flutter may differ significantly.
• Either atrial fibrillation or atrial flutter may be a substrate for sudden cardiac death for individuals with ventricular pre-excitation.
• Pediatric patients with Wolff-Parkinson-White disease have a higher incidence of sudden cardiac arrest compared to adults.

## Introduction

Risk stratification for the potential of sudden cardiac death in young patients with Wolff-Parkinson-White (WPW) syndrome remains a somewhat controversial and imprecise exercise. Although clinical parameters such as unexplained syncope or a family history of WPW may correlate with increased risk, most commonly the risk is estimated based on parameters observed during episodes of clinical tachycardia or variables measured during electrophysiology study (EPS).^1^^,^^2^ While variables such as the antegrade accessory pathway effective refractory period or the shortest paced cycle length with pre-excitation during atrial pacing are commonly used, the shortest pre-excited R-R interval (SPERRI) during atrial fibrillation is a generally considered the measurement that best defines the risk of sudden cardiac death, owing to rapid antegrade conduction resulting in ventricular fibrillation.^3^ In this report, we describe a patient with a high-risk accessory pathway whose SPERRI significantly shortened during sustained atrial flutter compared to SPERRI measurements in atrial fibrillation or other programmed stimulation parameters. The mechanism for this observation and the implications regarding risk stratification in young patients are discussed.

## Case report

**Figure 1:**
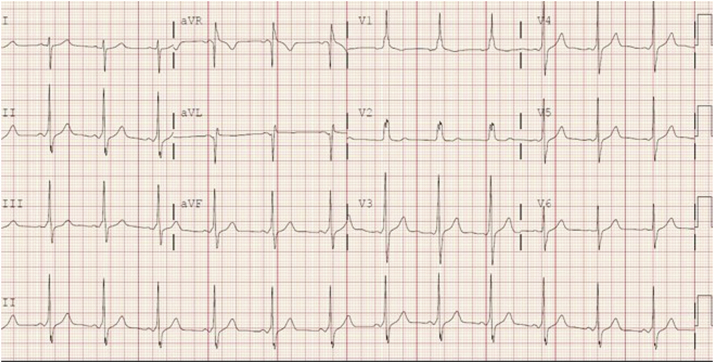
Baseline electrocardiogram tracing demonstrating ventricular pre-excitation of left anterolateral accessory pathway. The QRS complexes are significantly different from those with maximal pre-excitation in Figures 2 and 3.

This case involves a 16-year-old female patient with WPW and episodes of supraventricular tachycardia since the neonatal period. Owing to increasingly frequent and prolonged episodes of supraventricular tachycardia with symptoms including severe chest pain and near-syncope, she was referred for EPS and catheter ablation. Her baseline ECG demonstrated ventricular pre-excitation with a pattern consistent with left anterolateral accessory pathway (Figure 1).

**Figure 2:**
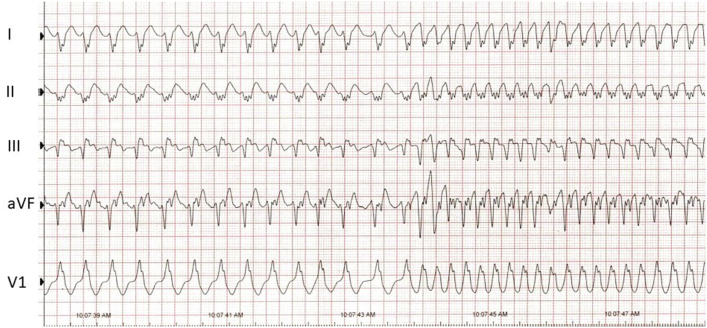
Surface electrocardiogram tracing of abrupt transition from atrial fibrillation to atrial flutter. Note the somewhat irregular pre-excited QRS complexes with abrupt transition to very rapid, regular, and maximally pre-excited QRS complexes.

**Figure 3:**
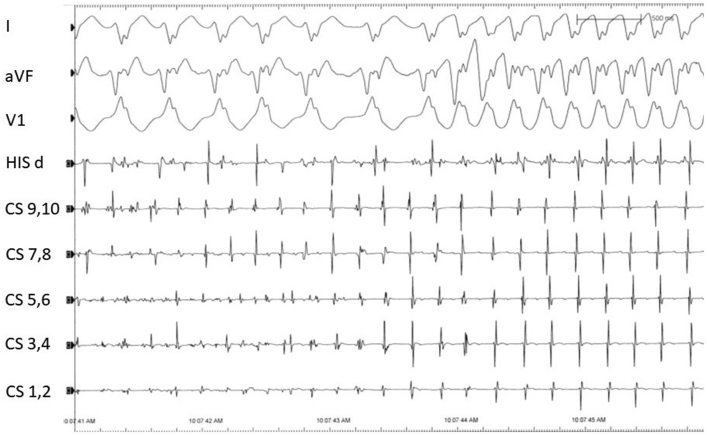
Intracardiac electrograms demonstrating the transition from atrial fibrillation with variable accessory pathway (AP) conduction to atrial flutter and consistent 1:1 AP conduction.

At diagnostic EPS, local ventricular pre-excitation with earliest activation was identified at the distal coronary sinus catheter. Ventricular pacing also demonstrated eccentric and nondecremental ventricular-atrial conduction at the anterolateral aspect of the coronary sinus. The antegrade accessory pathway effective refractory period was 270 ms with 600 ms cycle length drive train. There was persistent antegrade accessory pathway conduction with rapid atrial pacing at 260 ms. Induced orthodromic reciprocating tachycardia converted into atrial fibrillation with a single-interval SPERRI of 228 ms; however, the majority of R-R intervals were in the range of 350–400 ms (Figure 2). Atrial fibrillation then spontaneously converted to atrial flutter with a sustained ventricular cycle length of 195 ms owing to consistent 1:1 A-V accessory pathway conduction with maximally pre-excited QRS complexes (Figures 2 and 3) and loss of demonstrable cardiac output. Atrial flutter terminated spontaneously to normal sinus rhythm during preparation for DC cardioversion. Radiofrequency catheter ablation was then performed with complete elimination of bidirectional accessory pathway connection.

## Discussion

In patients with WPW, it is generally accepted that the risk of sudden death is related to the characteristics of the accessory pathway(s). The mechanism of sudden death is reported as atrial fibrillation with rapid antegrade accessory pathway conduction leading to ventricular fibrillation.^3^ However, this patient demonstrated high-risk accessory pathway characteristics with the SPERRI during atrial fibrillation ≤250 ms in 1 R-R interval only. Conversely, the SPERRI further shortened during atrial flutter with sustained 1:1 AP conduction at a cycle length of 195 ms.

For adult patients with WPW, high-risk is defined as the SPERRI during atrial fibrillation ≤250 ms, the presence of multiple accessory pathways, an accessory pathway refractory period ≤240 ms, or atrioventricular reentrant tachycardia precipitating pre-excited atrial fibrillation.^4^, ^5^ For children, catheter ablation is recommended for those with a SPERRI ≤250 ms, those with structural heart disease for which an arrhythmia may result in poor hemodynamics, or those who have developed ventricular dysfunction.^2^ However, it has also been reported that patients with life-threatening events may have accessory pathway properties that are not deemed high-risk and low threshold for ablation should be considered.^1^

Atrial flutter and atrial fibrillation are proposed to be related entities and may transform into one another, as demonstrated with our patient.^6^ This was associated with a significant decrease in the pre-excited R-R interval (195 ms) and cardiovascular collapse. The change in the SPERRI is consistent with concealed conduction during atrial fibrillation, resulting in variable prolongation of the accessory pathway refractoriness but with more uniform repolarization during atrial flutter allowing 1:1 conduction.^7^ This raises an important point when patients with WPW are deemed to have low-risk pathways based on EPS testing and the type of atrial arrhythmia (atrial fibrillation vs atrial flutter) that measurements are obtained. The transition of atrial fibrillation to atrial flutter with very rapid sustained conduction may offer a possible explanation for the higher incidence of sudden death in young patients with WPW syndrome compared to older patients.^8^

## Conclusion

Invasive risk stratification for pediatric patients with WPW is imperfect. The relationship of atrial fibrillation (with concealed conduction) and atrial flutter (absence of concealed conduction) may offer an explanation for sudden death or life-threatening events in children who have low-risk pathways by invasive electrophysiologic testing. Catheter ablation should be considered at the time of EPS if safe and feasible.
